# Successful Treatment of *Mycobacterium caprae* Infection in Two UK Cats

**DOI:** 10.1002/vms3.71180

**Published:** 2026-08-31

**Authors:** Emily Clark, Danielle Gunn‐Moore, Conor O'Halloran

**Affiliations:** ^1^ Small Animal Hospital School of Biodiversity One Health and Veterinary Medicine University of Glasgow Glasgow UK; ^2^ Royal (Dick) School of Veterinary Studies University of Edinburgh, Easter Bush Veterinary Campus Edinburgh UK

**Keywords:** cat, mycobacterial infection, raw meat based diet, treatment

## Abstract

A 4‐year‐old, male‐neutered Savannah cat (Case 1) and a 3‐year‐old female‐neutered British shorthair cat (Case 2) were each investigated for chronic coughing, lethargy and weight loss. Thoracic imaging revealed diffuse broncho‐interstitial changes and multifocal soft‐tissue nodules (Case 1), and two cavitating masses in the left lung (Case 2). Bronchoalveolar lavage cytology (BAL) in both cases identified macrophagic inflammation with acid‐fast bacilli and was culture (Case 1) or PCR (Case 2) positive for *Mycobacterium caprae*. The cats were treated with 11 months (Case 1) and 9 months (Case 2) of triple antimicrobial therapy comprising pradofloxacin (5 mg/kg orally once every 24 h), rifampicin (10 mg/kg orally once every 24 h) and azithromycin (15 mg/kg orally once every 24 h). Both made complete clinical recoveries and radiographic improvement in the thoracic changes were static for 2 months prior to cessation of therapy. Both cats remain clinically well 12 and 10 months after cessation of treatment, respectively.

## Graphical abstract

This article describes the first cases of successful treatment and long term resolution of clinical signs of two cats infected with *Mycobacterium caprae*. This suggests that the “triple antibiotics therapy” (rifampicin, azithromycin and pradofloxacin) used for other *Mycobacterium tuberculosis*‐complex infections in cats can be used safely and effectively in these infections. A larger number of patients undergoing treatment will be needed to fully evaluate treatment success rates and long term survival.

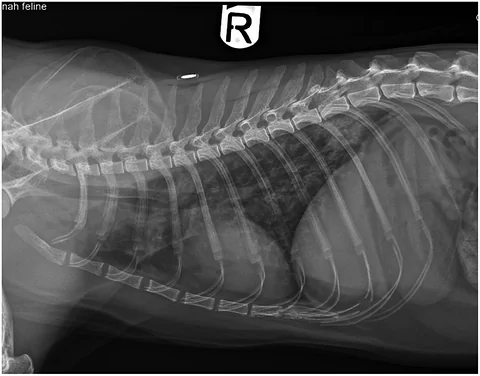

## 1 Case Description

### 1.1 Case 1

A 4‐year‐old male‐neutered Savannah cat was presented to his general practice veterinarian in Inverness, Scotland, for an intermittent cough and mild weight loss of 3 weeks’ duration. He had been a strictly indoor‐only cat since acquisition as a kitten. He had not been given routine antiparasiticides but was up to date with core vaccinations. He was fed a commercially available raw meat‐based diet (RMBD; poultry‐based [68%] with 27% supplemental beef offal).

On initial presentation, he was bright, alert and responsive. He weighed 5.18 kg with a body condition score (BCS) of 4/9. He had a heart rate of 168 beats per minute (bpm) and a respiratory rate of 40 bpm. No heart murmur was detected on thoracic auscultation, but he had slightly harsh lung sounds bilaterally, with an occasional wheeze. His temperature was normal, and the rest of the clinical examination identified no abnormalities.

He was initially treated with a 7‐day course of meloxicam (0.05 mg/kg once every 24 h [q24hrs] orally [PO] with food; Metacam, Boehringer) to which there was no response. Over the next 2‐weeks his cough became more persistent, and he became increasingly lethargic with a decreased appetite. He was anaesthetised, and thoracic radiographs were performed; these showed a marked broncho‐interstitial pattern. A blind bronchoalveolar lavage (BAL) was performed under the same anaesthesia, which was submitted for cytological evaluation as well as routine bacterial culture. The cytological examination revealed moderate neutrophilic inflammation and was negative on bacteriological culture.

Pending these results, the patient was empirically prescribed a 10‐day course of marbofloxacin (2 mg/kg PO q24hrs; Marbocyl, Vetoquinol) alongside meloxicam (as above). He showed no improvement, became more lethargic, hyporexic, and lost 6% of his body weight from the initial presentation.

**FIGURE 1:**
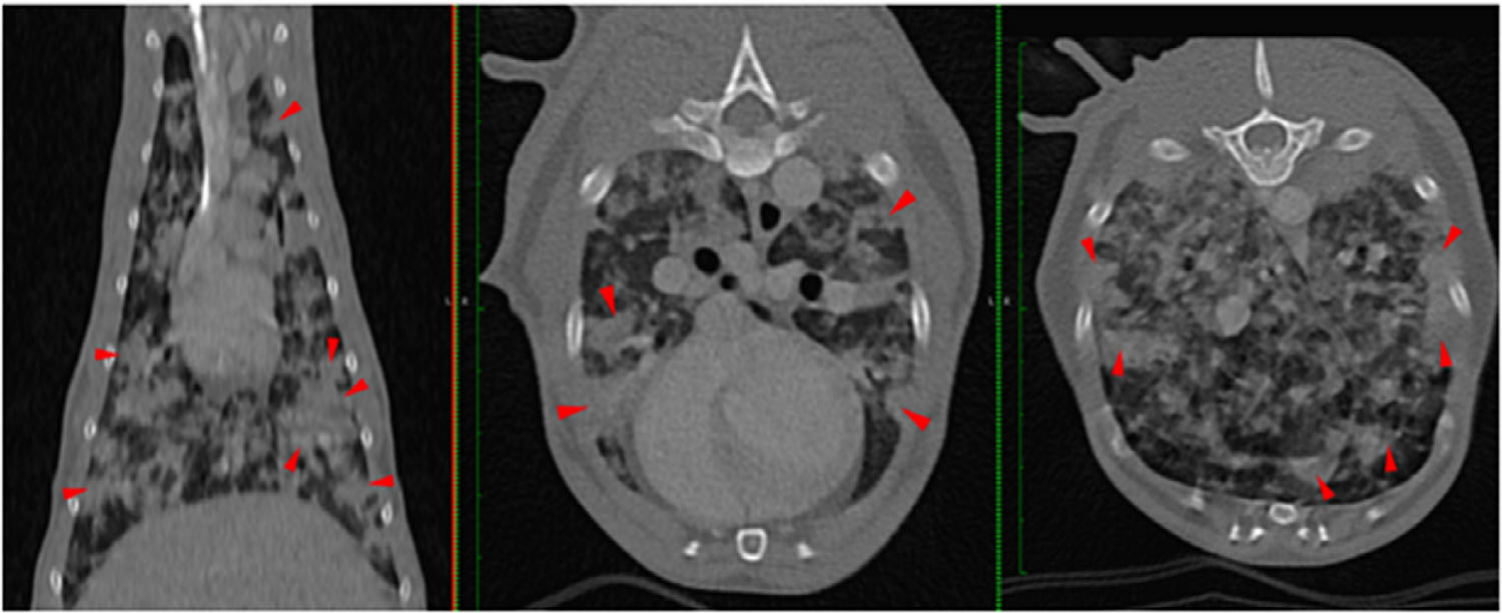
Three sections of the thoracic CT study of Case 1 (a 4‐year‐old male neutered Savannah cat) with a 2‐month history of chronic coughing, hyporxia and weight loss. The scan shows nodular to confluent soft tissue attenuating lesions with mild contrast enhancement within the pulmonary parenchyma (red arrowheads).

At this time, haematology and serum biochemistry profiles were unremarkable. Due to clinical progression, a thoracic computed tomography (CT) scan was performed, which revealed the pulmonary parenchyma to be severely, diffusely affected by nodular to confluent soft tissue attenuating lesions with mild contrast enhancement (Figure 1). The lesions were irregularly marginated and of various sizes, with the largest at the periphery of the lung parenchyma, with all lobes affected. The mediastinum was widened due to the presence of moderately enlarged mediastinal lymph nodes.

**FIGURE 2:**
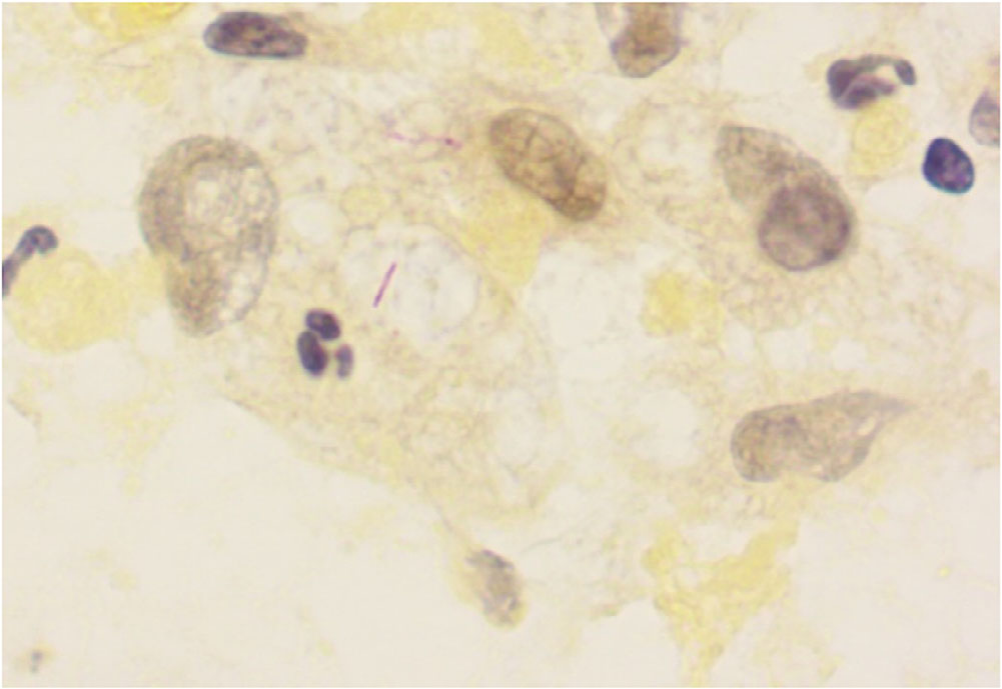
Cytological evaluation of a fine needle lung aspirate (Case 1) stained with Ziehl–Neelsen stain at 100× magnification. Within a macrophage cytoplasm is a single, acid‐fast, slender, rod‐shaped organism (red arrow) typical of *Mycobacterium* spp.

Considering the severity of the scan results, the patient was referred to the **REFERRAL INSTITUTION**. On presentation, the patient weighed 3.8 kg (30% loss since initial presentation) with a BCS of 2/9. Several fine‐needle aspirates were taken from areas of consolidated lung parenchyma and submitted for cytological examination, which identified severe neutrophilic and macrophagic (i.e., pyogranulomatous) inflammation. Subsequent slides were stained with the Ziehl–Neelsen (ZN) method and revealed small numbers of acid‐fast bacilli with slender, curved morphology, typical of mycobacteria (Figure 2). Samples were submitted for mycobacterial culture, which was positive for *Mycobacterium caprae*.

An oesophagostomy tube was placed while the patient was under anaesthesia to facilitate prolonged oral administration of the antibiotics by the owners. Triple antibiotic therapy was instigated comprising pradofloxacin (5 mg/kg PO q24hrs; Veraflox, Bayer), rifampicin (10 mg/kg PO q24hrs; generic) and azithromycin (15 mg/kg PO q24hrs; Zithromax, Pfizer). Liquid formulations were utilised to allow administration via the feeding tube.

The cat was hospitalised in an isolation facility at the **REFERRAL INSTITUTION** for a total of 8 days, with precautions in place as for all cats with mycobacterial infection (the number of in‐contact staff was kept to a minimum and wore personal protective equipment, including disposable gowns, gloves and N95 facemasks). Prior to discharge, a discussion about the zoonotic risks was had with the owners, and information was provided in writing for their consideration. Once the diagnosis of *M. caprae* was confirmed, relevant public health officials were notified and follow‐up contact was made by them with the owners, but no further testing or actions were deemed to be indicated. No staff at the Hospital were deemed to have performed any high‐risk procedures without adequate safety equipment, and so no further investigation or public health action was taken.

He was examined monthly whilst on treatment. Clinically, he improved rapidly and within a month had started to gain weight, with a notably decreased frequency of coughing.

**FIGURE 3:**
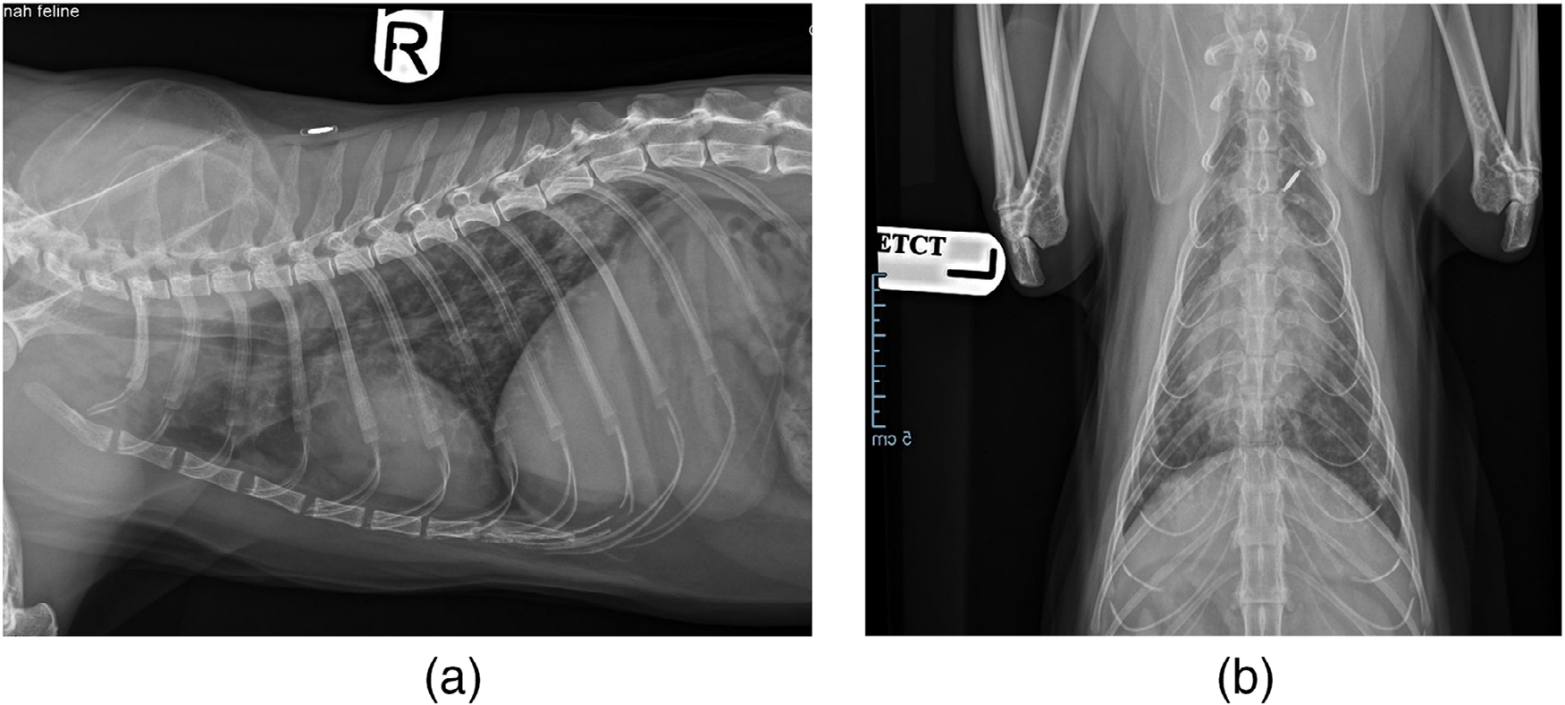
A right‐laterolateral (a) and dorsoventral (b) radiograph of the thorax of Case 1 at the time of discontinuing treatment, given static changes were present for the previous 3 months. These show a persistent diffuse nodular pattern.

Thoracic radiographs were repeated at 3‐monthly intervals. These showed mild improvement over the course of treatment but, even with full resolution of clinical signs, remained markedly affected (Figure 3).

When the lung changes on thoracic radiography were static for more than 2 months, 11 months after initiation, antibiotic treatment was stopped. The patient remains clinically normal at the time of writing, a year after cessation of treatment.

### 1.2 Case 2

A 3‐year‐old female neutered British Shorthair cat was presented to her general practice veterinarian in Hampshire, England, for a 1‐month history of a cough progressing to develop weight loss and fever. She had been a strictly indoor‐only cat since acquisition as a kitten, was not given routine antiparasiticides but was up to date with core vaccinations. She was fed a commercially available RMBD (the same product as Case 1).

On initial presentation, she was bright, alert and responsive. She weighed 3.67 kg with a BCS of 4/9. She had a heart rate of 132 bpm and an increased respiratory rate of 68 bpm. Her temperature was increased (39.9°C) whilst the rest of the clinical examination was unremarkable.

Initial investigations included haematology and serum biochemistry, including ionised calcium, which were unremarkable.

**FIGURE 4:**
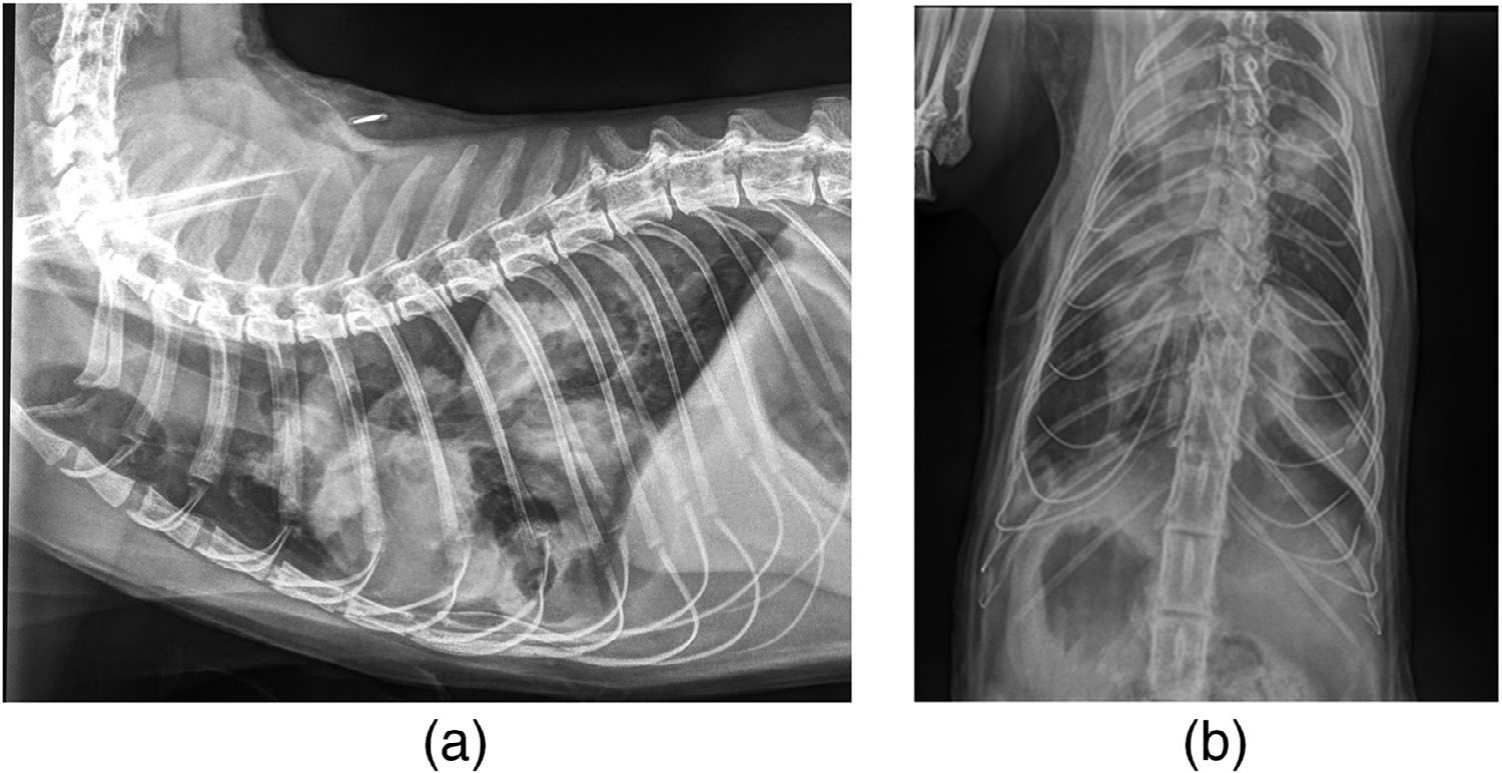
A right‐laterolateral (a) and dorsoventral (b) radiograph of the thorax of Case 2 at the time of diagnosis. These show a cavitating mass in the caudal left lung field with an additional large nodule in the left cranial lung (red arrows) and multifocal areas of dense consolidation diffusely through the remaining lung fields.

Thoracic radiographs revealed, most significantly, a cavitating mass in the caudal left lung field with an additional large nodule in the left cranial lung. There were multifocal areas of dense consolidation diffusely through the remaining lung fields (Figure 4).

Hospitalisation in an isolation facility was offered but declined by the clients. An in‐depth discussion about the zoonotic risks was had with the owners and information was provided in writing for their consideration. Once the diagnosis of *M. caprae* was confirmed, relevant public health officials were notified and follow‐up contact was made by them with the owners, the outcome of which is unknown. No staff were deemed to have performed any high‐risk procedures without adequate safety equipment and so no further action was taken.

The patient was anaesthetised and underwent a BAL. Cytology identified marked neutrophilic and macrophagic inflammation. The macrophages displayed epithelioid morphology with foamy cytoplasm and non‐staining ‘ghost’ bacilli intracellularly. Subsequent slides were stained with the ZN method and revealed small numbers of intracellular acid‐fast bacilli with slender, curved morphology, typical of mycobacteria. A sample was submitted for mycobacterial PCR testing (Leeds University Teaching Hospital, Mycobacterial Reference Laboratory) and was positive for DNA of the *Mycobacterium tuberculosis* complex (MTBC), and subsequent sequencing of the PCR product confirmed infection with *M. caprae*.

After consultation with the authors at the **REFERRAL INSTITUTION**, triple antibiotic therapy was instigated as for Case 1, also comprising pradofloxacin (5 mg/kg PO q24hrs; Veraflox, Bayer), rifampicin (10 mg/kg PO q24hrs; generic) and azithromycin (15 mg/kg PO q24hrs; Zithromax, Pfizer). Liquid formulations of each drug were used to facilitate patient compliance with long‐term daily medication. The patient was examined intermittently whilst on treatment. Clinically, she improved rapidly and within a month had started to gain weight, with a notably decreased frequency of coughing noted by 4 months of therapy.

**FIGURE 5:**
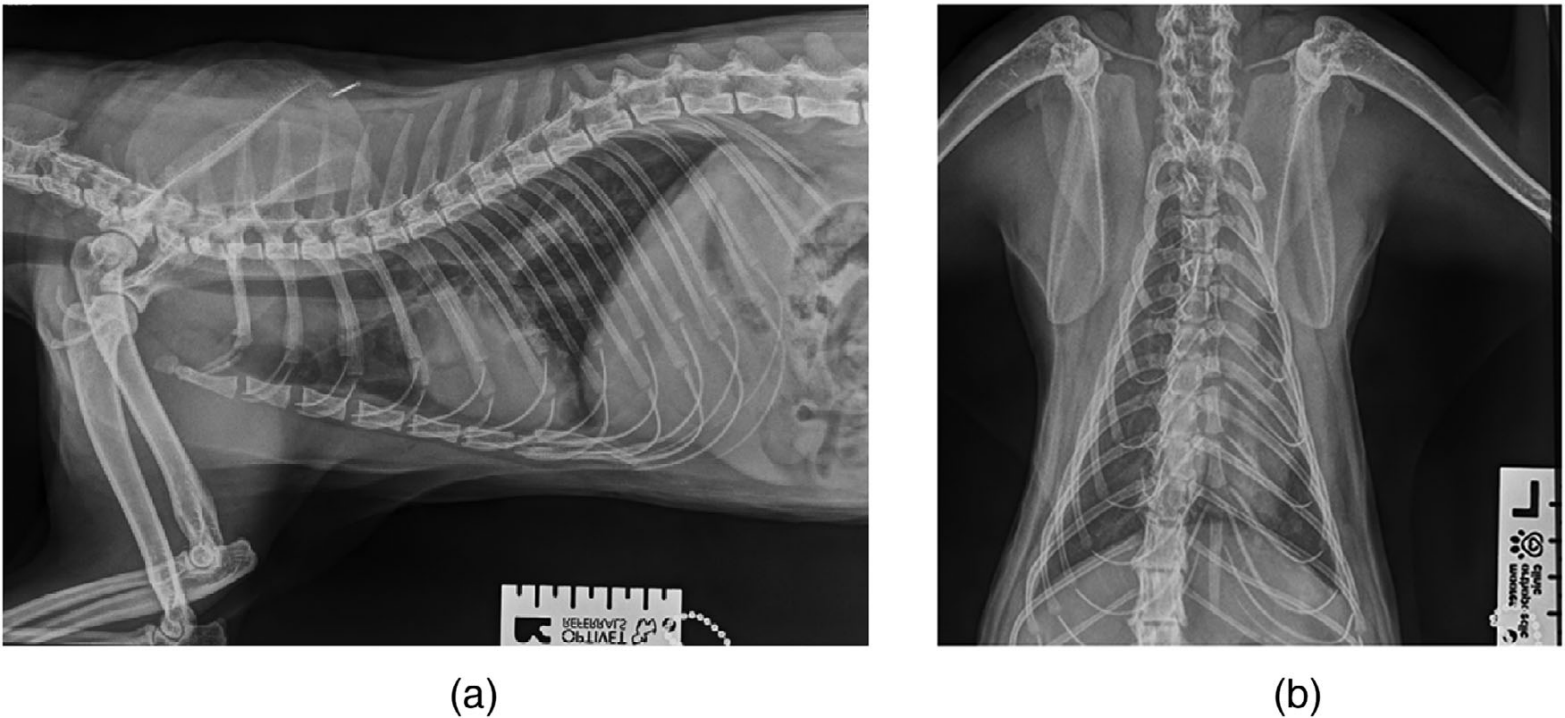
A right‐laterolateral (a) and dorsoventral (b) radiograph of the thorax of Case 2 at the time of discontinuing treatment, with static changes that were present for the previous 3 months but given marked improvement from initial diagnosis (Figure 4).

Thoracic radiographs were repeated after 5 months of therapy. These showed a significant improvement since the time of diagnosis but without complete normalisation (Figure 5). Antibiotic therapy was continued as above, and thoracic radiographs were repeated after an additional 3 months. These showed no change in the thoracic pathology and, given the patient was clinically well and had been on treatment for 9 months, therapy was discontinued. The patient remains clinically normal at the time of writing, 10 months after cessation of treatment.

## 2 Discussion

In the UK, feline MTBC infections are commonly caused by organisms *Mycobacterium bovis* or *Mycobacterium microti* (Gunn‐Moore, Brewer, et al. 2011). Infection with *M. caprae* has only recently been identified in domestic cats and has only been described in detail in one case that was examined post‐mortem (O'Halloran et al. 2024; Gola et al. 2025). This case report describes the successful management of *M. caprae* disease in cats for the first time.

The only large retrospective study looking at the success of treatment of mycobacterial infections in UK cats found that there was limited consistency in treatment protocols for these cases (Gunn‐Moore, Schock, et al. 2011). A significant number of these cats responded to antibiotic treatment, with 42% of them achieving complete remission. This was despite many receiving what would now be considered inappropriate treatment (e.g., short courses of fluoroquinolone monotherapy). Since that study was published 15 years ago, the authors have developed updated treatment strategies based on the advised treatment for human tuberculosis infections (NICE 2024), adapted for cats (O'Halloran and Gunn‐Moore 2017). This treatment consists of triple antimicrobial therapy with rifampicin at 10 mg/kg orally once daily, a fluoroquinolone (usually pradofloxacin in cats at standard once daily doses due to its excellent safety profile) and a macrolide/azalide (usually azithromycin due to its once daily dosing requirement which maximises patient compliance) for at least 3 months and for 2 months beyond the resolution of clinical signs and/or changes to thoracic radiographs (O'Halloran and Gunn‐Moore 2017). This approach has led to remission rates of approximately 80%. The same protocol was applied in the two cases treated in this report.

Substantial pulmonary pathological changes were identified in both patients, and it was not possible to reach radiographic resolution of the changes despite extended duration of antibiotic therapy. Instead, treatment was discontinued when the findings were consistent for at least 2 months, and no relapse in clinical signs has been noted. This situation appears to be analogous to a condition now increasingly recognised in humans as post‐tuberculosis lung disease (PTLD), which encompasses a spectrum of both structural and functional pulmonary abnormalities after successful anti‐mycobacterial therapy (Meghji et al. 2025; Al‐Hindawi et al. 2026; Fumagalli et al. 2026). Studies indicate that 18%–80% of treated patients have lasting residual lung damage which can lead to residual diagnostic imaging abnormalities (Meghji et al. 2025; Al‐Hindawi et al. 2026; Fumagalli et al. 2026). Stable radiographic changes in cats could therefore be seen as a reasonable therapeutic goal and could be used to determine the endpoint of therapy in cases of mycobacterial infection where radiographic changes are not expected to normalise based on their initial severity.

Both patients required prolonged therapy with a multidrug protocol daily, with frequent follow‐up appointments to reach remission of clinical signs. This required extensive financial and emotional investment by the caregivers and excellent patient compliance. In any cases of feline MTBC infection where these factors are not in place, careful consideration should be given to whether treatment is appropriate.

Initially, Cat 1 was treated with meloxicam for 7 days at the time of initial presentation. The reason for this is not immediately apparent, as the cat was not reported to have pyrexia or be in pain by the examining veterinarian. Different empirical medications may have altered the clinical course of the case, such as if an infectious component to the cough; for example, if *Mycoplasma* spp., was suspected, then doxycycline may have been considered, or if an inflammatory airway disease was suspected, then anti‐inflammatory corticosteroids could have been utilised. However, in this context, either of these drugs may have made the final diagnosis harder to reach or made the clinical signs more severe. Therefore, veterinarians need to consider empirical therapeutic choices carefully and discuss the potential benefits and risks with owners.

Given that *M. caprae* is a member of the MTBC, its potential transmission to humans warrants particular attention. Zoonotic transmission of *M. bovis* from cats has previously been documented, though the risk is considered very low (O'Connor et al. 2019). Transmission of *M. caprae* to humans has been identified in situations of proximity to infected goats (the reservoir host) but never from cats (Pérez de Val et al. 2025). Therefore, it was deemed reasonable to treat these cats, particularly as no in‐contact humans had any of the risk factors for zoonotic transmission, as previously described (O'Halloran and Gunn‐Moore 2017).

However, due to the potential for transmission and the development of antimicrobial resistance if dosing regimens are not adhered to, consideration should be given to euthanasia for patients with severe disease where compliance with appropriate medications cannot be provided.

Successful treatment can be aided by the placement of an oesophagostomy tube (as was performed in Case 1); this was in place for the entire duration of treatment to facilitate the administration of liquid medications by the owners, reducing stress to the patient and maintaining the owner–caregiver relationship.

## 3 Conclusion

As the first reported cases of successful treatment of *M. caprae* infection in UK domestic cats, this study contributes to the growing body of literature on mycobacterial infections in domestic animal populations. By raising awareness, it is hoped that this novel information can help to aid in successful patient management whilst mitigating the risks of zoonotic transmission.

## Author Contributions

E.C. was responsible for preparing the first draft of the manuscript. D.G.M. supervised the treatment of one of the cases and providing manuscript editing. C.O.H. supervised the study, was involved in the treatment of both cases and finalised the manuscript.

## Funding

The authors have nothing to report.

## Ethics Statement

The authors confirm that the ethical policies of the journal, as noted on the journal's author guidelines page, have been adhered to. The work described in this manuscript involved the use of non‐experimental (owned) animals with informed owner consent. Established internationally recognised high standards (‘best practice’) of veterinary clinical care for the individual patient were always followed. Ethical approval from a committee was therefore not required for publication.

## Conflicts of Interest

The authors declare no conflicts of interest.

## Data Availability

The data that support the findings of this study are available on request from the corresponding author. The data are not publicly available due to privacy or ethical restrictions.
